# Ureteritis Cystica Presenting with Atrophic Kidney: Report of a Case

**DOI:** 10.1100/tsw.2010.145

**Published:** 2010-08-03

**Authors:** Ayca Tan, Saime Unluoglu, Umit Bayol, Sehnaz Emil Sayhan, Deniz Altinel

**Affiliations:** Department of Pathology, Izmir Tepecik Training and Research Hospital, Izmir, Turkey

**Keywords:** atrophic kidney, ureter, ureteritis cystica

## Abstract

Ureteritis cystica is a rare proliferative condition that is found predominantly in the bladder, renal pelvis, and upper ureter. It may occlude the ureteral lumen and should be considered in the reasons for an atrophic kidney. A 65-year-old-female with a 2-year history of right flank pain that increased in the last 2 months was presented. Abdominal ultrasonography revealed right-sided atrophic kidney. Nephroureterectomy was performed. On the gross examination, along the ureter wall, there were numerous polyps, 0.5 cm in maximum diameter, protruding into the lumen. On the histopathological evaluation, ureteritis cystica and chronic pyelonephritis was detected. In conclusion, ureteritis cystica is a benign and indolent lesion that needs to be kept in mind among the causes of renal atrophy.

## INTRODUCTION

Ureteritis cystica (UC) is a silent pathology without any specific symptoms, most frequently detected incidentally during the evaluation of different conditions[1,2]. When UC has a polypoid growth, it may occlude the ureteral lumen, causing an atrophic kidney.

We present a 65-year-old female with UC in the upper ureter, diagnosed incidentally during the evaluation of an atrophic kidney.

## CASE REPORT

A 65-year-old female reported to the urology policlinic with complaints of recurrent urinary tract infections and right flank pain for 2 years. Ultrasonography of her abdomen and pelvis demonstrated unilateral multiple renal calculi and atrophic kidney. Right nephroureterectomy was performed. The right nephroureterectomy specimen, with ureter 5 cm in length and kidney 13 × 7 × 4.5 cm, was received. On the cut surface of the kidney, the pelvicaliceal system was dilated, the renal papillae were blunted, and the renal cortex was markedly thinned. In the proximal part of the ureter, several translucent cyst-like formations were observed. Some of them appeared pedunculated with a maximum diameter of 0.5 cm, and they contained a yellowish gelatinous material hanging from the inner coat into the canal of the ureter.

Microscopic examination of the kidney showed tubular thyroidization and atrophy, interstitial fibrosis, and patchy lymphoplasmacytic inflammatory infiltrate consistant with chronic pyelonephritis and atrophy (Fig. 1). Microscopic examination of the ureter showed an intact cystic structure lined by benign urothelium with intact umbrella cells, most consistent with cystitis cystica (Figs. 2–4). No goblet cells, dysplasia, or malignancy was identified.

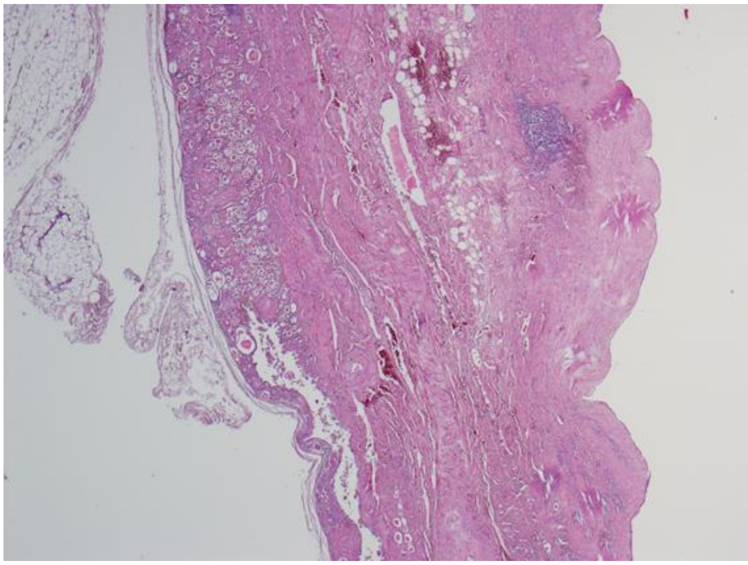

**FIGURE 1.** Tubular thyroidization and atrophy, interstitial fibrosis, and patchy lymphoplasmacytic inflammatory infiltrate in the kidney

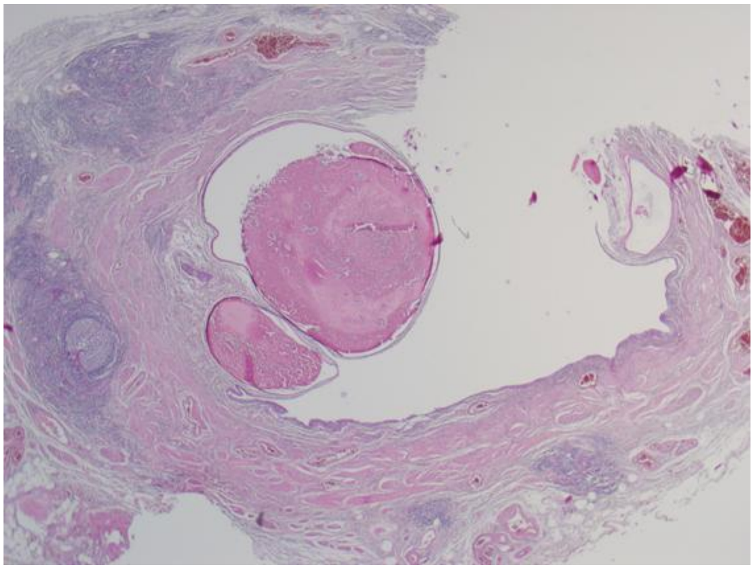

**FIGURE 2.** Small submucosal epithelial-lined cysts, protruding into the lumen, surrounded by nonspecific inflammatory infiltrate

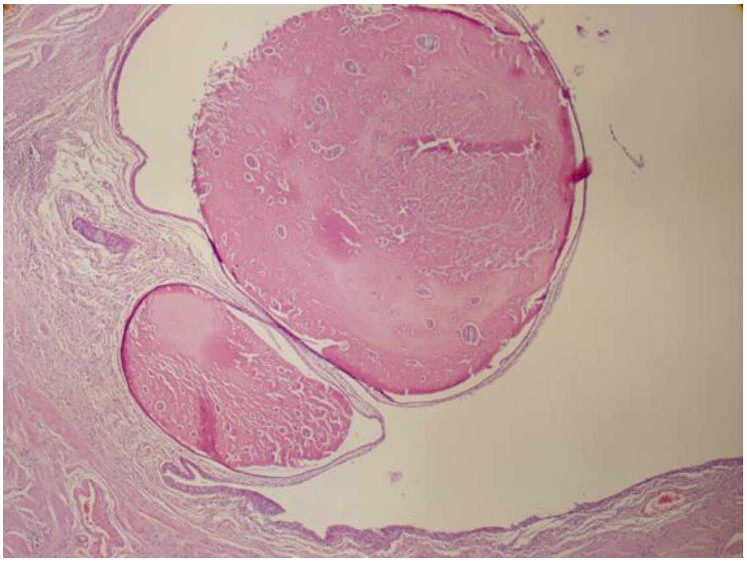

**FIGURE 3.** Intact cystic structure lined by benign urothelium.

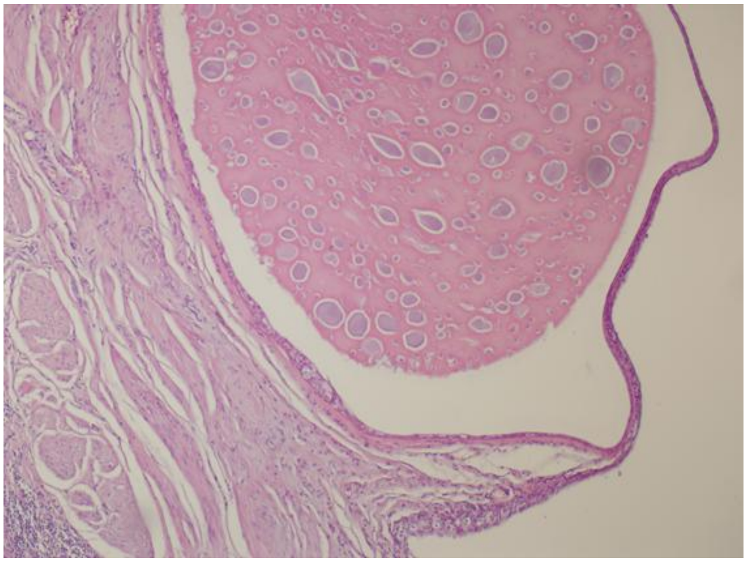

**FIGURE 4.** The cysts' lumens were filled with a colloid like material.

According to the findings, the present case was diagnosed as hydronephrosis, chronic pyelonephritis, and ureteritis cystica as an incidental finding.

## DISCUSSION

UC was reported first by Morgagni and was first described by Richmond and Robb[2,3,4]. From the time UC was first reported, approximately 200 cases have been declared in the literature. UC is a rare, benign, proliferative condition characterized by multiple cysts and filling defects in the urothelium[2]. UC is characterized with numerous small submucosal epithelial-lined cysts due to chronic inflammation[1]. UC may also be associated with chronic urothelial irritation. Nephrolithiasis and urinary tract infections have been demonstrated as the causes of UC in the literature[2,4]. In one study, the formalin treatment of cyclophosphamide-induced hemorrhagic cystitis has been found to cause UC[5]. In the present case, the reason of UC was probably due to the multiple staghorn calculi in the caliceal system, which provoked the recurrent urinary tract infections.

UC is most commonly found in the renal pelvis, upper portion of the ureter, and bladder[6]. The current lesion was located in the upper ureter.

UC is most frequently detected incidentally during the evaluation of different conditions[2]. Typically, the cysts are not large enough to cause complete obstruction, or dilation of the pelvis or ureter by themselves. In the literature, there are limited UC cases presented as a pedunculated polyp[1]. The polyp may serve as a valve system that eventually may cause kidney atrophy, as in the present case.

Grossly, the ureter showed several fluid-filled vesicles, some of which appeared pedunculated and contained a yellowish gelatinous material, on the urothelial surface, hanging from the inner coat into the canal of the ureter.

Microscopically, there are numerous, small, submucosal, epithelial-lined cysts representing cystic degeneration of epithelial cell nests within the lamina propria (cell nests of von Brunn), formed by downward proliferation of buds of surface epithelium that have become detached from the mucosa associated with a diffuse inflammatory process**.** The cysts' lumens were filled with a colloid-like material in which red blood corpuscles and degenerated epithelial cells were seen. The lining of these cysts, as a rule, consists of a single or double layer of flat epithelial cells, resembling endothelial cells somewhat, surrounded by a dense fibrous tissue envelope.

In the current case, UC and nephrolithiasis occurred together. Even though the case was evaluated as nephrolithiasis clinically, UC was the main cause of the atrophic kidney. As known, UC is determined incidentally due to urinary infections and nephrolithiasis. In the present case, obstructive UC was the significant cause for the atrophic kidney, which makes it unique in the literature.

In conclusion, UC, although an incidental diagnosis, should be considered in the differential diagnosis of renal atrophy when it is presented as a pedunculated polyp.
